# The Relationship between Trop-2, Chemotherapeutic Drugs, and Chemoresistance

**DOI:** 10.3390/ijms25010087

**Published:** 2023-12-20

**Authors:** Tomas Koltai, Larry Fliegel

**Affiliations:** 1 Hospital del Centro Gallego de Buenos Aires, Buenos Aires 2199, Argentina; tkoltai@hotmail.com; 2 Department of Biochemistry, Faculty of Medicine, University of Alberta, 347 Medical Science Bldg., Edmonton, AB T6G 2H7, Canada

**Keywords:** drug resistance, notch signaling, oxaliplatin, tamoxifen, Trop-2

## Abstract

Trop-2 is a highly conserved one-pass transmembrane mammalian glycoprotein that is normally expressed in tissues such as the lung, intestines, and kidney during embryonic development. It is overexpressed in many epithelial cancers but is absent in non-epithelial tumors. Trop-2 is an intracellular calcium signal transducer that participates in the promotion of cell proliferation, migration, invasion, metastasis, and probably stemness. It also has some tumor suppressor effects. The pro-tumoral actions have been thoroughly investigated and reported. However, Trop-2’s activity in chemoresistance is less well known. We review a possible relationship between Trop-2, chemotherapy, and chemoresistance. We conclude that there is a clear role for Trop-2 in some specific chemoresistance events. On the other hand, there is no clear evidence for its participation in multidrug resistance through direct drug transport. The development of antibody conjugate drugs (ACD) centered on anti-Trop-2 monoclonal antibodies opened the gates for the treatment of some tumors resistant to classic chemotherapies. Advanced urothelial tumors and breast cancer were among the first malignancies for which these ACDs have been employed. However, there is a wide group of other tumors that may benefit from anti-Trop-2 therapy as soon as clinical trials are completed.

## 1. Introduction

Drug resistance in cancer patients represents a notorious limit in the search to cure cancer, and importantly, when it occurs, it leaves oncologists with no further resources aside from palliative treatment [1]. Following Goldie’s words, it “remains the thorniest obstacle in developing improved systemic therapies for disseminated cancer”. Drug resistance, along with multidrug resistance have been the object of intensive research since 1958, when Burchenal and Holmberg were the first to study the short-lived remission achieved with anti-metabolites in the treatment of leukemia [2]. In 1973, Keld Danø proposed the theory of drug extrusion as the main mechanism of drug resistance, although he did not identify the membrane proteins involved [3]. This identification did not take place until the 1990s, when multidrug resistance transport proteins were discovered that remove chemotherapeutic compounds from within cancer cells [4,5,6].

Drug/multidrug resistance is not only the product of drug efflux proteins; many other mechanisms can evoke resistance to chemotherapy. This includes extracellular acidity which prevent the entry of drugs that are weak bases. Other causes of resistance include low vascular perfusion and increased interstitial pressure, which decrease drug accessibility to tumors. Mutation of driver genes to which a drug was addressed can cause resistance as can decreased expression of membrane proteins that transport chemotherapeutic drugs such as gemcitabine into cancer cells. The desmoplastic reaction of the stroma can cause chemoresistance, and alteration of the genetic profile of the patient can, for example, lead to temozolomide resistance in glioblastoma. Resistance to apoptosis is another cause of chemoresistance, though these phenomena are beyond the scope of this review [7,8,9].

Three transmembrane proteins often get all the credit for drug resistance. They belong to the ATP Binding Cassette family (ABC) and are P-glycoprotein (P-gp also known as MDR1), Multidrug Associated Resistance Protein1 (MRP1), and Breast Cancer Resistance Protein (BCRP) [10,11]. These proteins export drugs, thus preventing them from reaching their intracellular target. However, these three transport proteins are not the only transmembrane proteins that can play an important role in drug resistance. Other transmembrane proteins play a role in chemoresistance through different mechanisms. This is the case with a protein often known as Trop-2. Trop-2 has many other names, such as trophoblast antigen 2, tumor-associated calcium signal transducer 2 (TACSTD2), trophoblast cell surface antigen 2, gastrointestinal tumor-associated antigen-1 GA733.1, 40 KD glycoprotein, pancreatic carcinoma marker protein GA733-1, membrane component chromosome 1 surface marker 1 M1S1, epithelial glycoprotein-1, EGP-1, CAA1, Gelatinous Drop-Like Corneal Dystrophy GDLD protein, TTD2, gp50/TROP-2 [12], membrane component surface marker-1. From here on, we shall use the abbreviation Trop-2, which is the most widely used in publications. In this contribution, we review the characteristics of Trop-2, the role of Trop-2 in chemoresistance, and the mechanism of action in cancer. We also suggest strategies to target Trop-2 and improve outcomes in resistance to chemotherapy.

## 2. Trop-2 Structure

**Figure 1:**
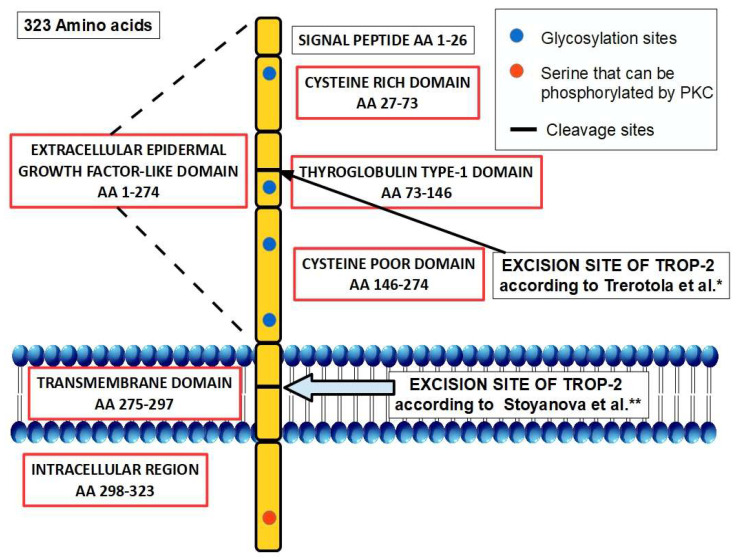
Structure of Trop-2 in the cell membrane [14]. Trop2 is a one-pass transmembrane protein that has 323 amino acids and is divided into a large N-terminal extracellular portion, a single-pass transmembrane portion, and a short C-terminal portion. The extracellular portion is composed of three domains: (1) a small N-terminal cysteine-rich domain (Figure 2), (2) a thyroglobulin type-1 domain, and (3) a cysteine-poor domain. The N-terminal cysteine-rich domain has a signal sequence between amino acids (AA) 1 to 26. The extracellular portion has four glycosylation sites, and the intracellular portion has a serine (Ser 303) that can be phosphorylated by protein kinase C. Two different Trop-2 excision sites have been described: Trerotola et al.* [15] report a cleavage site between amino acids 87 and 88 that is produced by ADAM10, while Stoyanova et al.** [16] locate another cleavage site in the intramembrane portion between amino acids 285 and 286 that is produced by a γ-secretase.

Trop-2 is coded by the intronless gene Tacstd2 in chromosome 1p32 [13]. The structure of Trop-2 is described in Figure 1.

**Figure 2:**
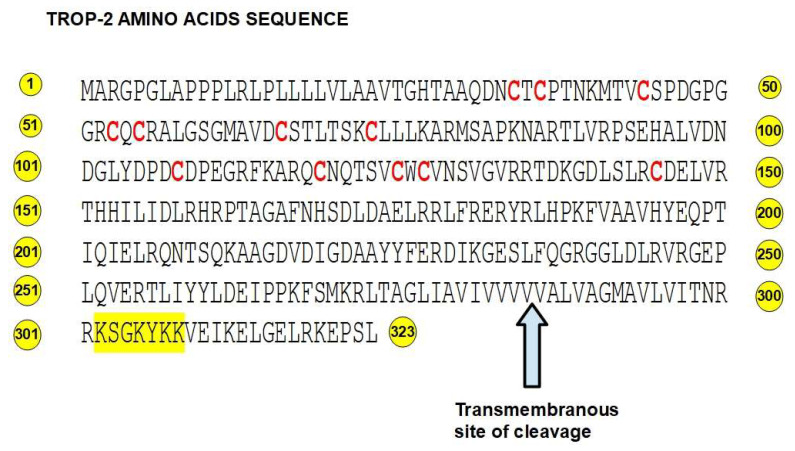
Amino acid sequence of Trop-2. 12 cysteines are in red. The protein kinase C phosphorylation and PIP2 binding regions are highlighted in yellow. The arrow shows the site of cleavage in the transmembrane region.

**Figure 3:**
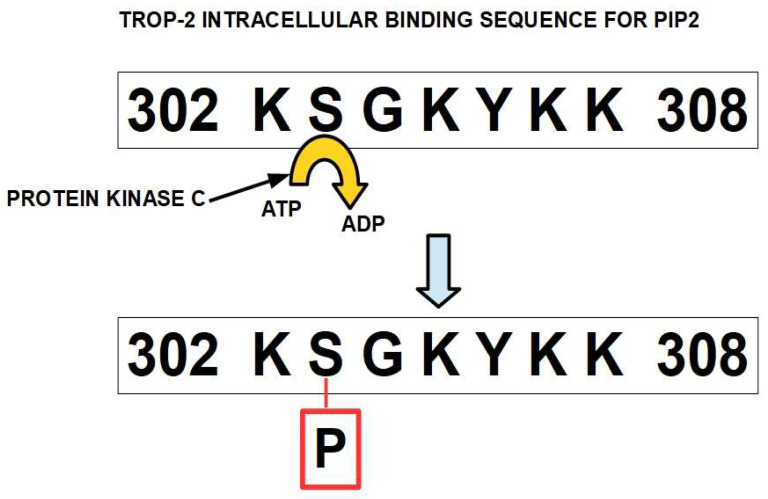
Binding sequence for PIP2 in the intracellular domain of Trop-2 between residues 302 and 308 [19]. Protein kinase C can phosphorylate serine 303.

Trop-2 belongs to the EpCAM (epithelial cell adhesion molecule) family of proteins and is highly conserved in mammals. Trop-2 and EpCAM (also known as Trop-1) have 49% sequence identity [17]. The main differences are in the intracytoplasmic domains. Importantly, both have pro-tumoral effects. These findings led to the theory that Trop-2 had its origin in the retroposition of EpCAM [18] or in the retroposition of a common ancestor. In this process, transcribed and spliced mRNA is reverse transcribed and then inserted into a gene to form the retrogene. Trop-2’s intracellular domain has a specific binding site for the phosphatidylinositol 4,5-bisphosphate (PIP2)-binding sequence that overlaps with a protein kinase C phosphorylation site [19] (Figure 3). This PIP2-binding sequence is a key regulatory element in the effects of the protein.

## 3. Functions of Trop-2

Trop-2 and EpCAM are regulators of cell-cell adhesion mechanisms through their extracellular region, which resembles an epidermal growth factor-like structure. Trop-2 and EpCAM are cell adhesion molecules that are structurally different from the other four known molecules, integrins, cadherins, selectins, and immunoglobulin CAMs [20]. Trop-2 participates in tight junction and basal membrane integrity [21]. Although it is not fully known, it has been shown that Trop-2 has many functions in the cell that are not related to cell adhesion, such as proliferation, invasion, and stemness [22,23].

## 4. Trop-2 Expression

Trop-2 is a cell surface protein that is minimally, or not at all, expressed in normal adult epithelial cells. It was originally found as a cell surface marker in trophoblastic cells, in 1981 by Lipinsky et al. [24]. Trop-2 is a marker of stem cells in adult murine tissues [25], and plays a role in the development of some organs, such as kidneys [26].

Trop-2 is overexpressed in many epithelial tumors [27], such as lung [28], pancreas [29,30], gall bladder [31], breast (the three subtypes) [32,33], urothelial and urinary bladder [34,35], prostate [36], salivary gland [37], hepatic [38], colorectal [39,40], and papillary thyroid [41] cancers. There was no Trop-2 expression found in melanoma, small cell lung cancer, granulosa cell tumor of the ovary, Leydig cell tumor, GIST, clear cell renal carcinoma, seminoma, pheochromocytoma, muscular, and neural tumors [42]. In general, Trop-2 is not expressed in hematological malignancies. These findings confirm that the expression of Trop-2 is limited to epithelial tumors [43].

Trop-2 expression was also found to be higher in hormone receptor-positive/HER2-negative tumors and triple-negative breast cancer (TNBC). In many tumors with overexpressed Trop-2, this glycoprotein can be considered an oncoprotein that deserves to be targeted. This seems to be particularly valid in breast cancer, where Trop-2 gene silencing suppresses TNBC cell growth in vitro and in vivo [44].

## 5. Trop-2 Pro-Tumoral Mechanisms of Action

**Figure 4:**
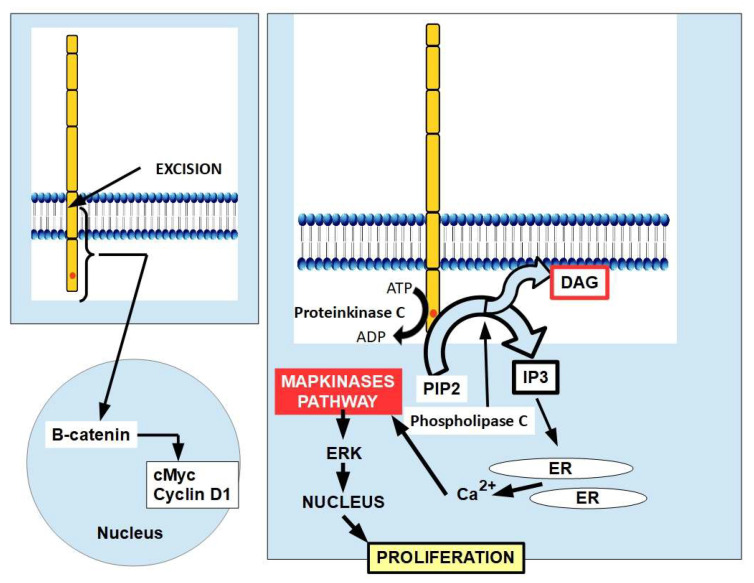
Mechanisms of action of Trop-2. The left panel shows the excision of Trop-2, releasing the transmembrane and intracytoplasmic portions that migrate to the nucleus, activating the pro-proliferative β-catenin pathway, leading to cMyc and cyclins D and E activation. The right panel shows the phosphorylation of Trop-2 by protein kinase C [46,47,48], which is essential for its pro-tumoral activity. The right panel also shows the partition of PIP2 into DAG and IP3 through the actions of phospholipase C. Both PIP2 and DAG act as second messengers. IP3 releases calcium from intracellular stores in the endoplasmic reticulum (ER). Increased intracellular calcium levels activate MAPK, which in turn increases levels of phosphorylated ERK1 and ERK2. ERK1 and ERK2 are important mediators of cell cycle progression, angiogenesis, cell proliferation, cell invasion, and metastasis. Intracellular calcium also activates the NF-κB pathway, which is involved in stimulation of cell growth, and the Raf signaling pathway, which is essential for the upregulation of *FOXM1*, one of the most overexpressed genes in human solid tumors. DAG activates protein kinase C, which stimulates tumor cell proliferation, invasion, and migration. This figure is based on references [49,50,51,52,53].

An essential step of Trop-2 activation consists of Trop-2 intramembrane cleavage, which leads to the formation of two segments:

(a) An extracellular segment that is shed and may stay in the membrane or can be incorporated into the cytoplasm. Its further effects are not clear [16].
(b) An intracellular segment that migrates to the nucleus, where it activates the β-catenin pathway which induces pro-tumoral effects through cMyc and cyclin D1 activation (Figure 4, left panel).

Cleavage is produced by a complex initially formed by TACE (TNF-α converting enzyme), PS1 (presenilin 1), and PS2 (presenilin 2), and finally cleaved by a gamma secretase. All these processes take place inside the cell membrane [16].

Trop-2 can contribute to tumor progression. Phosphorylation of intracellular serine 303 by protein kinase C allows phospholipase C to excise PIP2 into IP3 and diacylglycerol (DAG) [45]. IP3 then migrates towards the endoplasmic reticulum (ER), releasing ionic calcium (Ca^2+^). DAG remains on the inner surface of the membrane (Figure 4).

## 6. Trop-2 Signaling

The initial research showed that the Trop-2 membrane protein was a signal transducer with an intracellular portion that could regulate a second messenger represented by ionic calcium (Ca^2+^) [19]. It has been suggested that phosphatidylinositol 4,5-bisphosphate (PIP2) may play a role in Trop-2 phosphorylation and calcium signal transduction. This assumption is based on the finding that the Trop-2 intracytoplasmic PIP2-binding sequence overlaps with a protein kinase C phosphorylation site [19,54]. Furthermore, nowadays we know that Trop-2 is also involved in other signaling systems such as β catenin and Notch (Figure 4 and Box 1).

Cytoplasmic Ca^2+^ is very low under normal conditions because it is sequestered into the endoplasmic reticulum for internal storage. When Trop-2 is phosphorylated in its intracellular segments, phosphoinositides (probably from the membrane) are excised, and the IP3 that is produced causes Ca^2+^ release from the ER, which in turn activates the MAPK pro-proliferative pathway. Calcium release into the cytoplasm also activates the NF-kB pathway. (For a detailed explanation of Trop-2 signaling, see Cortesi et al. [55]).

## 7. Trop-2 and Drug Resistance

There is evidence that Trop-2 can be involved in promoting drug resistance, and this effect can be reduced by Trop-2 inhibition. Furthermore, chemotherapy can, in some cases, increase Trop-2 expression. For example, tamoxifen treatment significantly increased Trop-2 expression in breast luminal cancer cell lines [56].

Below, we outline 14 different types of evidence that support a role for Trop-2 in chemotherapy resistance.

► Varughese et al. [57] found that Trop-2 was highly expressed in chemotherapy-resistant ovarian cancer cell lines (expressed in 83% of 50 ovarian serous carcinoma specimens). They also demonstrated that these tumor cells were highly sensitive to a humanized anti-Trop-2 monoclonal antibody in vitro.

► Jordheim et al. [58] suggested that Trop-2 is potentially involved in a paradoxical growth-promoting effect of oxaliplatin. When oxaliplatin was administered to a xenograft model of HCT-116 human colon cancer cells, Trop-2 was overexpressed.

► Han et al. [59] present a case of a 74-year-old woman with widespread therapy-resistant uterine serous carcinoma who had previously failed multiple immunotherapy and chemotherapy regimens. This patient showed an excellent response to IMMU-132 (which contains an anti-Trop-2 antibody and a topoisomerase inhibitor) with a 66% reduction of target lesions by RECIST criteria and a duration of response beyond 10 months.

**Figure 5:**
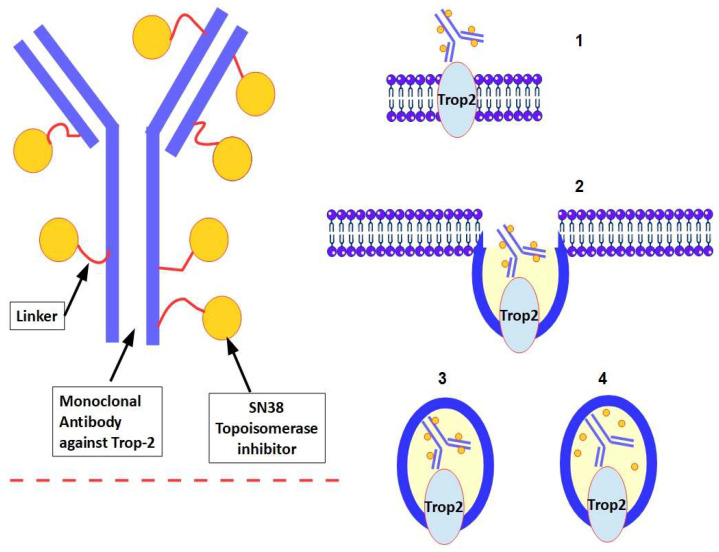
Left panel: structure of sacituzumab govitecan. The right panel shows the mechanism of penetration of the ADC into the cancer cell. (1) The monoclonal antibody binds Trop-2; (2) and (3) internalization of the ADC next occurs; (4) SN-38 is released by hydrolysis of the linker. Finally, SN-38 is discharged into the cell (not shown in the figure). Lysosomal acidity also plays a role in SN38 release [96]. 50% of the monoclonal antibody against Trop-2 is internalized within one hour [91,97]. Furthermore, the monoclonal antibody against Trop-2 has anti-tumor activity per se [98], in addition to that of SN38.

► Trop-2 is widely expressed in urothelial carcinomas. In a Phase I/II study of IMMU-132 in patients with urothelial cancer, Faltas et al. [60] reported six patients with metastatic, platinum-resistant urothelial carcinoma who were treated with sacituzumab govitecan (IMMU-132), a Trop-2-directed monoclonal antibody and topoisomerase inhibitor drug conjugate (see below Figure 5 for further details). The median number of previous therapies was 3 and 50% of patients had a clinically significant response.

► Another Phase II study of patients with SCLC evaluated IMMU-132 in heavily pretreated metastatic SCLC patients, including patients who were chemoresistant or chemosensitive to first-line chemotherapy. In this study, 60% of patients demonstrated tumor shrinkage when compared to baseline CTs, with a 34% clinical benefit rate [61].

► AKT is a therapeutic target for treating malignant tumors. Trop-2 is a predictor of tumor response to AKT inhibitors. Trop-2 if closely correlated with AKT levels and activation in tumor cells. Downregulation of Trop-2 blocked the tumor response to AKT blockade [62].

► Lung cancer cells rendered chemoresistant could be re-sensitized by knocking down Trop-2. In vivo experiments demonstrated that Trop-2 knockdown tumor xenografts displayed a slower growth rate than controls [63].

► Sacituzumab govitecan [64] combined with ABC transporter inhibitors restored the toxicity of SN38 (a powerful topoisomerase inhibitor) in breast and gastric cancer cells that were resistant in vitro and in vivo [65].

► Sun et al. [66] reported a possible mechanism involved in Trop-2-induced chemoresistance. They found that Trop-2 bound IGF2R, promoting the Akt pathway and inducing resistance to gefitinib in NSCLC through two mechanisms: decreased apoptosis and extracellular matrix remodeling.

►Trop-2 downregulation increased the cisplatin sensitivity of cervical cancer cell lines [67]. The number of apoptotic cells was significantly increased with cisplatin treatment of Trop-2 siRNA-treated cells compared with controls.

► Different Trop-2 expression among diverse prostate cancer cell lines indicates relapse potential. Androgen-sensitive prostate cancer cell lines with high Trop-2 levels had an increased ability to re-grow after docetaxel chemotherapy. When Trop-2 was down-regulated, these cells lost their ability to re-grow [68].

► Kuai et al. [69] used two different types of gastric cancer cells to show that Trop-2 promotes multidrug resistance. In one, Trop-2 was knocked down, while in the other it was overexpressed. When these cells were used to generate xenografts, those with Trop-2 knocked down had a significantly greater sensitivity to cisplatin. On the other hand, those with Trop-2 overexpression were highly resistant to treatment. The authors found that Trop-2 decreased the proliferative inhibition induced by chemotherapeutic treatments and decreased cell apoptosis. Trop-2 silencing increased chemotherapeutic effects. Importantly, “the expression of MRP1 was decreased after Trop-2 inhibition, and overexpression of Trop-2 promoted the expression of MRP1”. Activation of the notch pathway by Trop-2 was found to be involved in mediating the chemoresistance.

► Trop-2 expression tends to increase in some malignant cell lines exposed to oxaliplatin, but not in all lines of colorectal cancer [70]. The authors proposed Trop-2 expression as a marker of oxaliplatin resistance.

► Varughese et al. [57] showed that chemo-resistant ovarian carcinoma cell lines could be re-sensitized by inhibiting Trop-2 with a monoclonal antibody, RS7-3G11.

## 8. Mechanisms of Trop-2-Induced Drug Resistance

A mechanism by which Trop-2 may act to increase drug resistance was suggested in a study by Cho et al. They showed that Notch signaling regulates the expression of MRP1 [71]. This leads to a possible pathway that seems to be operative in Trop-2-mediated drug resistance. Supporting this theory is also evidence showing that Notch downregulation can decrease drug resistance in prostate cancer and ovarian cancer cells [72,73]. This mechanism is shown in Box 1 [20,43,74].

Box 1 **One of the mechanisms of Trop-2 chemoresistance.**

**    Trop-2--------------→ Notch --------------→ MRP1**

Another study by Sun et al. [66] determined that Trop-2 induced gefitinib resistance in NSCLC. In this case, the mechanism found was Trop-2 binding IGF2R, which in turn promoted IGF2-IGF1R-Akt signaling, which increased gefitinib resistance. Silencing Trop-2 and IGF1R simultaneously allowed gefitinib-induced decreases in proliferation (Box 2). These studies give some insights into the mechanism of Trop-2 resistance, but more work in this area is needed to clarify the mechanisms in the various cell types.

Box 2 **Trop2 stimulates IGF1R-Akt signaling which increases gefitinib resistance.**

**    Trop-2-------→ IGF2R -------→ AKT-------→ Cell survival**

## 9. Trop-2: Controversial Effects

Despite all the above-mentioned evidence [75], Trop-2 seems to exert anti-tumoral effects as well. Sin et al. [76] found that Trop-2 has tumor-suppressive effects in cervical cancers by inhibiting IGF1R and Alk receptors. Knocking down TACSTD2 expression (the Trop-2) in breast cancer MCF7 cells increased proliferation [77]. In another study, it was also found that head and neck squamous cell carcinomas lost Trop-2 expression when they became resistant to gefitinib [78]. There is no clear explanation for these controversial findings. However, we may theorize that Trop-2 participation in chemoresistance is tissue- or tumor-specific.

## 10. Sp1 Is a Transcription Factor That Induces Trop-2 Expression

Specificity protein 1 (Sp1) is a transcription factor that promotes the transcription of many pro-tumoral and some anti-tumoral proteins. However, it can be considered mainly as a pro-tumoral factor. Sp1 promotes Trop-2 expression. Sp1 knockdown impedes Trop-2 expression [79]. The trop-2 promoter area contains CpG islands with four Sp1 binding sequences. Sp1 transcription factor can be downregulated with non-steroidal anti-inflammatory drugs such as celecoxib and particularly tolfenamic acid [80,81,82,83]. Importantly, there is evidence that tolfenamic acid can reduce chemo and radio resistance [84].

## 11. Targeting Trop-2

Conventional chemo and radiotherapy or targeted therapy did not change the Trop-2 expression in pre-treatment samples compared with post-treatment in patients with non-small lung cancer [85]. Therefore, the idea that conventional cancer treatment may decrease Trop-2 expression has no experimental basis. This leads to the idea that further targeting is required, since Trop-2 is an important cancer promoter in some tumors. In this regard, two approaches can be considered:

(a) Trop-2 direct targeting via Trop-2 neutralization.
(b) Using Trop-2 as a mechanism to deliver drugs such as sacituzumab and govitecan into the cells.

**(a) Trop-2 neutralization with antibodies: antibody-dependent cellular cytotoxicity**

Several studies have shown that applying antibodies against Trop-2 to malignant cells expressing Trop-2 results in anti-tumoral effects through antibody-dependent cellular cytotoxicity (ADCC) [86,87,88,89,90,91,92], as well as other mechanisms [93]. 

**(b) Sacituzumab govitecan**

As mentioned above, another approach is to use antibodies against Trop-2 to target drugs, specifically tumors. Sacituzumab govitecan is one such antibody-drug conjugate (ADC) directed against Trop-2. Sacituzumab is a humanized monoclonal antibody that specifically recognizes Trop-2. The drug conjugated is SN-38, which is an inhibitor of topoisomerase I. SN-38 binds to the antibody via a hydrolyzable linker. Seven to eight molecules of SN-38 bind to each antibody molecule (Figure 5). Sacituzumab govitecan has been approved as a monotherapy for the treatment of adult patients with some types of breast cancer. It is for cases of unresectable or metastatic breast cancer that are hormone receptor (HR) positive and HER2 negative. Patients should have previously received treatment based on endocrine therapy and would have had at least two additional systemic therapies in the advanced stage [94,95].

Sacituzumab govitecan binds to cancer cells that express Trop 2 and is internalized with the subsequent release of SN-38 from the hydrolyzable linker (Figure 5). SN-38 interacts with topoisomerase I and prevents topoisomerase I-induced DNA single-strand breaks from re-ligating. The resulting DNA damage leads to apoptosis and cell death.

## 12. Discussion

Trop-2 is a membrane glycoprotein overexpressed in many epithelial tumors and involved in different signaling pathways. It was found to be a predictor of poor patient survival [99,100,101,102], a higher risk of tumor recurrence [103], and metastasis [104]. However, an important distinction must be made. Ambrogi et al. [33] found that Trop-2′s specific location in breast cancer cells was an important prognostic marker: functional Trop-2 membrane distribution was an unfavorable marker for overall survival, while an intracellular location had the opposite effect on cell survival. This finding was later confirmed by further study [105]. Nevertheless, at this point, there is no doubt that Trop-2 is a pro-tumoral glycoprotein [106,107,108] and that the tacstd2 gene can be considered an oncogene.

The relationship between Trop-2 and drug resistance and the mechanisms involved are poorly known. Evidence shows that Trop-2 expression in cancer participates in drug resistance against pharmaceuticals such as tamoxifen, oxaliplatin, cisplatin, and trastuzumab. Although there is indirect evidence, it is not conclusive for Trop-2 participation in multidrug resistance. The two known resistance mechanisms are AKT activation and Notch signaling. A third possible mechanism seems to be tamoxifen-induced Trop-2 expression through the transcription factor TFEB [56]. There are probably also other mechanisms that are not yet known.

Cancer stem cells seem to play an essential role in chemoresistant tumor relapse [109,110]. It was proposed that stemness represents a driver for tumor and cancer relapse [111,112]. On the other hand, Trop-2 expression has been found to be associated with stem cells in prostate cancer [16,25,113]. Therefore, evidence indicates, at least in prostate cancer, that Trop-2 may be associated with chemoresistant relapse through increased stemness.

The success obtained with the antiTrop-2/SN38 conjugate in patients unresponsive after intensive treatment with conventional chemotherapies [114] is probably the best proof of concept of Trop-2 participation in chemoresistance.

## 13. In Summary

(1) Many tumors showing drug resistance overexpressed Trop-2, and importantly, they were highly sensitive to Trop-2 immunologic neutralization.
(2) Trop-2 was able to reverse the effects of oxaliplatin, promoting growth instead of apoptosis.
(3) Tamoxifen and cisplatin induced the overexpression of Trop-2.
(4) Tamoxifen-resistant cells had a higher expression of Trop-2 in breast cancer cells.
(5) Inhibiting Trop-2 in drug-resistant patients could make them more sensitive to chemotherapy drugs.
(6) Trop-2 knockdown increased the cytotoxic effects of many chemotherapeutic drugs.
(7) Trop-2 increased the expression of MRP1, but we did not find a direct relationship between Trop-2 and other MDR proteins.
(8) Trop-2 overexpression is a predictor of tumor response to Akt inhibitors.
(9) Inhibition of the specificity protein-1 (Sp-1) can reduce Trop-2 expression.
(10) Notch signaling seems to be involved in Trop-2-dependent resistance.

## 14. Conclusions

Trop-2 is expressed in many tumors but not in all. In many cases, it was suggested to have an indirect role in membrane protein-mediated chemotherapy resistance. There is evidence showing that Trop-2 down-regulation can decrease/inhibit chemoresistance. Furthermore, a new generation of drugs have been created that seem to be effective in the treatment of chemotherapy-resistant tumors. These are the antibody drug conjugates that combine the potent cytotoxicity of chemotherapy with the antigen-specific targeted approach of antibodies into one single molecule.

Although there is some controversy that needs to be clarified, Trop-2 deserves further research as a potential target to antagonize multidrug resistance.

## Data Availability

Not applicable.
